# Why is individual reproduction in *Drosophila* flies stochastic?

**DOI:** 10.3389/fgene.2012.00324

**Published:** 2013-01-30

**Authors:** V. N. Novoseltsev, J. A. Novoseltseva

**Affiliations:** Institute of Control Sciences, Russian Academy of SciencesMoscow, Russia

Reproduction is essential in studying of aging and senescence in fruit flies as for the resources devoted to egg-laying are subtracted from the total resources of the organism and thus intensive reproduction shorten the life span of an individual. According to prevailing opinion, the stochastic nature of spatial and temporal distribution of external factors leads to randomness of reproductive patterns in fly populations in the wild. Environments are often not constant, but can vary stochastically. The amplitude of variability in environmental conditions influences organismal responses (Boyce et al., 2006; Boggs, 2009). It was believed that the random character of egg-laying can be studied with stochasticity of reproductive behavior (Markow, 1996). Nonetheless, the question of why real egg-laying patterns are stochastic was never asked until 2003 (Novoseltsev et al., 2003b).

To analyze random individual reproductive patterns for the first time it was proposed that *Drosophila* females should be studied by a three-stage non-random approximation embracing the time interval from hatching to death. The analysis included maturation (when eggs are ripened in ovarioles), adult stage (when eggs are laid in a maximal rate), and the senescence stage, when energy shortages produce an exponential decrease of this rate.

This means that the total “stochasticity” in reproductive process was reflected by a set of random parameters adjusted individually for each pattern. Five parameters were used—the moment of the first egg laid was *t*_0_, duration of constancy interval of egg-laying *T*, time constant of exponential tail τ, reproductive capacity *RC*, and life span *LS* (Novoseltsev et al., 2004).

This three-stage approximation technique is the only one which produces the whole pattern description starting with an individual's hatching and ending at death. In this, it is different from the approximations related to various segments of the reproductive process. For example, in Muller et al. (2001) it is shown that individual reproduction experiences exponentially decline in advanced ages, and in Rauser et al. (2003) the assertion is expressed that eggs laying is not reduced to zero in the end of life, but stabilizes at some low level.

Still, the description of individual reproductive patterns contains two types of stochasticity. Slow changes in reproduction, connected with age alterations, are reflected in the “skeleton” of the pattern, but also there exist fast, day-to-day variations in egg-laying, reflected in the tooth-like graph imposed on the three-stage skeleton.

Here, we discuss a hypothesis about the mechanisms that lead to the emergence of the second type of the randomness in the pattern, and on this basis we construct the model of egg production *in silico*. Then this model is used to study heterogeneity in resource allocation and causes of death in fruit flies.

The death of a fly usually occurs at advanced ages when reproductive process has been completed. This is a death caused by senescence. However, in the case of a large share of resource for reproduction and a lack of resources for somatic maintenance, death may occur earlier. In this case, the fly is still in the full swing of egg-laying and death from reproductive overload occurs.

## Individual reproduction pattern

**Figure 1:**
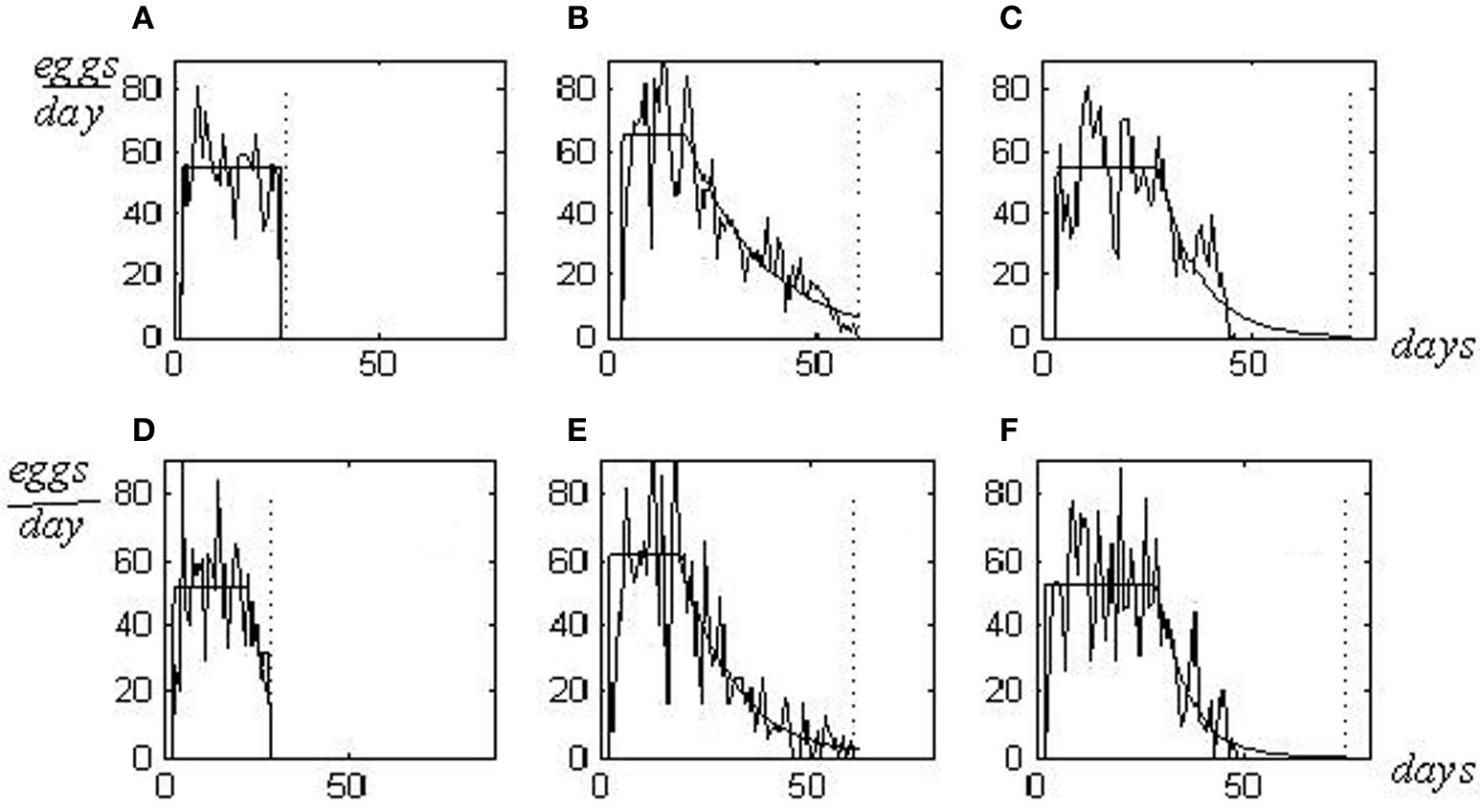
**Experimental and *in silico* reproductive patterns in *D. melanlgaster*.** Patterns for *s-, m*-, and *l*-flies are shown as shortened, medium and long lives as related to the reproduction periods [**(A–C)**—experimental patterns for flies 226, 29, and 2; Arking (1987) population database; **(D–F)**—the correspondent *in silico* patterns]. Death caused by reproductive overload is shown (*s*-flies, **A** and **D** plates), as well as deaths from senescence [*m*- and *l*- flies, plates correspondingly **(B,C** and **E,F**)]. Parameters of regularly approximated patterns for to, *T, RC, LS* and ½ are as follows: 2, 24, 55, 27, 0.02 in *s*-flies; 3, 16, 66, 60, 18 in *m*-flies; 3, 16, 25, 55, 74, 10 in *l*-flies. The *in silico* values of the parameters *a, T* and *LS* are 50, 19, 29 in *s*-flies; 65, 20, 60 in *m*-flies, and 55, 26, 75 in *l*-flies. Vertical dotted lines—the life spans.

Individual reproduction pattern represents the graphical picture of a sequence of numbers showing the quantity of eggs laid day-to-day by a fly, from hatching until death. Three individual reproductive patterns, which correspond to three various phenotypes in resource allocation (*s-, m*-, and *l*-patterns), are shown in Figures 1A–C. Letters *s-, m*-, and *l*- (from *shortened, medium*, and *long*) define the life span as related to the reproduction period. *S*-flies have a large energy resource devoted to reproduction but a small resource assigned for somatic maintenance. Their lives are interrupted when egg production continues at a high rate and hasn't diminished yet. Death caused by reproductive overloading arises so that flies die prematurely, not achieving the senescence stage (panel **A**). *M*-flies have a balanced resource allocation and their reproductive potential is realized practically entirely **(B)**. In *l*-flies, balance is displaced to a large somatic resource; thus they continue to live after completing reproduction **(C)**. Females of *m*- and *l*-types die from senescence whereas *s*-flies die from reproductive overload (Novoseltsev et al., 2005).

Individual reproductive pattern is a random process in the formation of which at least three body systems are involved: (1) the energy balance system, (2) the system of dietary protein processing in the yolk-protein, and (3) the system of ovarioles in which eggs mature before laying.

The first two systems define regular changes in reproduction (the structure of the pattern). Energy relations define the “skeleton,” determining moments of the transition from stage to stage—the beginning of the period of maturity (egg-laying) and the beginning of the senescence period. The ovarioles system provides fast (day-to-day) fluctuations, reflecting the number of eggs laid per day.

The energy source of the organism *E*(*t*) is defined as:

$$E(t)=\int_{0}^{t}[P_{1}(\tau )-P_{2}(\tau )]d\tau ,$$

where *P*_1_ stands for the daily energy intake, and *P*_2_ is the total daily energy expenditure: *P*_2_(*t*) = *P*_s_ + *P*_r_(*t*). Here *P*_s_ = const—daily somatic energy consumption, and *P*_r_(*t*)—daily consumption of energy for reproduction (proportional to the per day number of eggs laid).

Daily intake of energy *P*_1_(*t*) is subject to the aging process. After a period of constancy its quasi-exponential decline starts with maximum daily energy intake *P*_1MAX_ and oxidative vulnerability β_1_.

The energy balance is responsible for the slow alterations in the oviposition and creates a “skeleton” pattern. While the possibility of the energy system is such that the allowable production of energy *P*_1MAX_ exceeds operating costs, processes in the body flow constantly. But at a time *t* = *T*, when the daily consumption of energy *P*_s_ + *P*_r_(*t*) becomes greater than its arrival, production and egg-laying process begin to decline. Death occurs when energy resource is exhausted, *E*(*LS*) = 0.

A simplified model of the system which processes protein into yolk-protein is described as follows. Let *a* = const stands for the intake of protein from outside (a unit of measurement being the amount of protein required for the formation of a single egg), and *x*(*k*)—the amount of protein in the body at the *k*-th day. Further, let *n*(*k*)—protein intake in the system, *x*(*k*)—the amount of protein in it, and *L*—its capacity (the maximum amount of protein, which can be processed simultaneously). The whole protein *n*(*k*), received by the system at the *k*-th day, turns into a yolk-protein and the next day goes into ovarioles. All ovarioles are identical and each of them can mature to *M* eggs simultaneously.

If the quantity of yolk-protein in the body denoted as *y*(*k*) = *n*(*k* − 1), the balance of protein can be determined by the equation *x*(*k*) = *x*(*k* − 1) + *a* − *n*(*k*), and the processing of the protein in yolk-protein is described as *x*_*y*_(*k*) = *x*_*y*_(*k* − 1) + *n*(*k* − 1) − *n*(*k* − 2).

The resulting yolk-protein enters the ovarioles and fills them in turn. Admission of new eggs in the ovarioles is determined by the condition *x*_ov_(*j, k*) ≤ *M*, where *x*_ov_(*j, k*) is a number of eggs in the *j*-th ovariole at the *k*-th day. This condition, once satisfied, begins as soon as the process of laying eggs forms “free place” in the ovariole. Immediately after that, a new portion of the eggs is loaded. The quantity of yolk-protein *y*(*k*), entering the ovarioles, depends on the values of *a, L*, and the initial amount of protein in the body *x*(0).

The rate of the yolk-protein admission to the system of ovarioles, *y*(*k*), determines the rate of egg production. If a lot of protein comes with the food (*a* > *L*/2), the plateau level *RC* is determines by the possibilities of the processing system. Otherwise (*a* ≤ *L*/2), *RC* depends on the rate of protein income. Thus, *RC* = min (*L*/2, *a*). This model considers variant *a* ≤ *L*/2 to avoid a necessity of analysis of forced oscillations.

These relations are valid only until the beginning of the aging process: a decline in egg production starts after time *T*. According to the model (Novoseltsev et al., 2003b), which reflects the oxidative damage theory, the aging of the system of the protein into the yolk-protein processing occurs in the same way as that of the energy system. Thus, these processes take place under the condition *x*_*y*_(*k*) ≤ *N*(*k*), where: *N*(*k*) = *L*, if 0 ≤ *k* ≤ *T*. Otherwise, $N(k)=L.\exp[-\beta_{2}\int_{k-T}^{k}P_{2}\left(\tau \right)d\tau ]$, if *k* > *T*.

Here β_2_ is the coefficient of oxidative vulnerability in the protein processing system. It was previously shown that in the *Drosophila* organism the protein processing system ages faster than the power system (Novoseltsev et al., 2003a). Thus, β_2_ > β_1_ in the model.

## Stochastic nature of eggs laying

It can be assumed that the randomness of the process of oviposition in nature is primarily concerned with the uneven provision of energy to numerous ovarioles. At early stages of development, when the future ovarioles are only starting to form, genetic noise plays an important role. Thus, ovarioles receive a variety of power equipment, and eggs in different ovarioles mature at different times ξ.

To form a random reproductive pattern, we define the duration of the maturation process at random: ξ is the integer uniformly distributed in the interval [*p* + 1, *p* + *q*]. The value of *p* determines the day of the first egg laid, and *q*, the time delay from the first mature egg to the last one. Maturation itself lasts for several days. Thus, for *p* = 0, *q* = 4 the maturation of eggs in the ovarioles takes from 1 to 4 days.

Let us denote the total number of ovarioles in the body with *J*, and yolk-protein intake in the *j*-th ovaryole at the *k*-th day with *m*(*j, k*). If there is a lot of yolk-protein in the body, first ovarioles are filled completely, i.e., each of them receives a portion of exactly *M* oocytes: *m*(*j, k*) = *M*. The number of eggs laid from the *j*-th ovarioles for the *k*-th day, is denoted as *s*(*j, k*). Then the number of eggs in the ovarioles

$$x_{\text{ov}}(j,k)=x_{\text{ov}}(j,k-1)+m(j,k-1)-s(j,k-1).$$

Ripe eggs are laid from the ovariole: *y*_2_(*j, k*) = *s*(*j, k*). The total number of eggs laid on the *k*-th day, *s*(*k*), obtained by summing the eggs laid from all *J* ovarioles. Daily energy consumption devoted to reproduction on the *k*-th day, *P*_r_(*k*), is defined by the number of eggs laid on this day: *P*_r_(*k*) = α·*s*(*k*). Here *a* is the energy cost of laying of a single egg. The aging of ovarioles is not provided in the model.

Changing the setting *P*_1MAX_ allows the reproduction of patterns of *s-, m*-, and *l*-flies shown above in Figures 1A–C. These patterns *in silico* are presented in Figures 1D–F.

## Discussion

The assumption is made above that the stochastic nature of oviposition in fruit flies is completely determined by internal properties of the organism, although environmental factors might have a certain impact.

In formalizing the random nature of eggs maturation in ovarioles the following approach is possible. It assumes all ovarioles the same, and the number of days required for each portion of maturing oocytes to turn into ripened eggs is random. In this case, each portion of eggs laid from the ovariole needs a different amount of time to mature. The process of laying eggs is random due to the fact that every day the eggs are laid from different ovarioles having different maturation time. This assumption allows the construction a model which reproduces *in silico* the entire cycle of egg production and laying. The resulting pattern is virtually indistinguishable from the ones in real experiments, and the model allows confirmation of the early results of the death of a fly caused by reproductive overload.
